# Optimising recovery of DNA from minimally invasive sampling methods: Efficacy of buccal swabs, preservation strategy and DNA extraction approaches for amphibian studies

**DOI:** 10.1002/ece3.70294

**Published:** 2024-09-12

**Authors:** R. Martin, K. E. Mullin, N. F. D. White, N. Grimason, R. Jehle, J. W. Wilkinson, P. Orozco‐terWengel, A. A. Cunningham, S. T. Maddock

**Affiliations:** ^1^ Faculty of Science and Engineering, School of Life Sciences University of Wolverhampton Wolverhampton UK; ^2^ School of Science, Engineering and Environment University of Salford Salford UK; ^3^ Amphibian and Reptile Conservation Bournemouth UK; ^4^ Cardiff School of Biosciences Cardiff UK; ^5^ Institute of Zoology, Zoological Society of London London UK; ^6^ School of Natural and Environmental Sciences Newcastle University Newcastle upon Tyne UK; ^7^ Department of Life Sciences The Natural History Museum London UK; ^8^ Island Biodiversity and Conservation Centre University of Seychelles Victoria Seychelles

**Keywords:** anurans, DNA recovery, genetic sampling techniques, genomics

## Abstract

Studies in evolution, ecology and conservation are increasingly based on genetic and genomic data. With increased focus on molecular approaches, ethical concerns about destructive or more invasive techniques need to be considered, with a push for minimally invasive sampling to be optimised. Buccal swabs have been increasingly used to collect DNA in a number of taxa, including amphibians. However, DNA yield and purity from swabs are often low, limiting its use. In this study, we compare different types of swabs, preservation method and storage, and DNA extraction techniques in three case studies to assess the optimal approach for recovering DNA in anurans. Out of the five different types of swabs that we tested, Isohelix MS‐02 and Rapidry swabs generated higher DNA yields than other swabs. When comparing storage buffers, ethanol is a better preservative than a non‐alcoholic alternative. Dried samples resulted in similar or better final DNA yields compared to ethanol‐fixed samples if kept cool. DNA extraction via a Qiagen™ DNeasy Blood and Tissue Kit and McHale's salting‐out extraction method resulted in similar DNA yields but the Qiagen™ kit extracts contained less contamination. We also found that samples have better DNA recovery if they are frozen as soon as possible after collection. We provide recommendations for sample collection and extraction under different conditions, including budgetary considerations, size of individual animal sampled, access to cold storage facilities and DNA extraction methodology. Maximising efficacy of all of these factors for better DNA recovery will allow buccal swabs to be used for genetic and genomic studies in a range of vertebrates.

## Graphical abstract

In this multiple‐case study, we tested a comprehensive set of factors impacting DNA recovery from buccal swabs, including the type of swab, storage type and conditions and DNA extraction method. Based on our results, we are able to propose recommendations for improved DNA recovery, taking into consideration budget and field condition constraints.

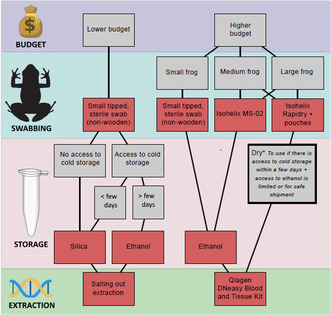

## 1 INTRODUCTION

Genetic analyses are increasingly becoming a key component of conservation management strategies (Fuentes‐Pardo & Ruzzante, 2017; Wayne & Morin, 2004). In line with the fragility of populations warranting conservation, increased awareness of animal welfare and the requirement of animal research to implement the ‘3 Rs’ of *replacement*, *reduction* and *refinement*, there is a growing demand that non‐invasive or minimally invasive sampling techniques be used when studying wildlife (Lefort et al., 2019). The use of toe clips and tail tips has until recently been commonplace when sampling live amphibians and reptiles for genetic studies (Funk et al., 2005; Gamble, 2014), but these raise ethical concerns about animal health and welfare. Having been used for decades as both a method of capture‐mark‐recapture and a genetic tissue sampling, there is now an increased body of literature identifying negative impacts of toe clipping on amphibians and reptiles (e.g. Beaupre et al., 2004; Funk et al., 2005; Liner & Smith, 2007; McCarthy & Parris, 2004; Perry et al., 2011; Zemanova, 2020). Recent advancements in molecular techniques have meant that DNA recovery has improved at the extraction phase, and many downstream approaches now require less DNA template than previously. We are now at a stage where many population‐level molecular studies can and should be conducted using less‐invasive techniques for DNA collection that do not require toe clipping or the removal of other tissue.

Such less‐invasive techniques could include the use of buccal, skin or cloacal swabs. Buccal and cloacal swabs are well documented as effective minimally invasive DNA sampling techniques for many taxa of amphibians (Urodela: Balázs et al., 2020; Pidancier et al., 2003; Poschadel & Möller, 2004; Gymnophiona: Adamson et al., 2016; Maddock et al., 2014; Anura: Ambu & Dufresnes, 2023; Broquet et al., 2007; Goldberg et al., 2003; Müller et al., 2013; Pidancier et al., 2003, Poschadel & Möller, 2004). Skin swabbing for host DNA collection has not been as successful for frogs and caecilians (Maddock et al., 2014; Müller et al., 2013; Ringler, 2018) as it has been for newts (Pichlmüller et al., 2013; Prunier et al., 2012; Ward et al., 2019), due to challenges such as high contamination (potentially due to the presence of skin alkaloids and microbiota, Ringler, 2018). Skin swabbing, however, has been widely used for the detection of DNA of amphibian skin pathogens such as *Batrachochytrium dendrobatidis* (e.g. Hudson et al., 2019; Hyatt et al., 2007). Skin swabs are also commonly used to identify the amphibian skin microbiome (e.g. Kueneman et al., 2014). While skin swabs are considered a less suitable option for recovering DNA compared to buccal swabs, it is worth noting that buccal swabbing may not always be possible, especially for smaller species/individuals with small mouths. In these circumstances, skin swabs might be the only non‐destructive alternative.

While the use of swabs for DNA collection is recognised as an alternative to more invasive methods of tissue sampling such as toe clipping, they inherently capture less genetic material. Generally, skin swabs have not always yielded enough DNA for genotyping, with significantly lower DNA quality and quantity compared to toe clips (Ringler, 2018) or buccal swabs (Prunier et al., 2012). This lower DNA yield has traditionally limited the potential of using DNA recovered from swabs for some downstream analyses. Maximising DNA yield and purity is thus important to increase the range of applications of DNA collected from swabs. Additionally, studies reporting DNA quantification of buccal swabs in amphibians using the Qubit™ are scarce, with most studies measuring DNA with less accurate photometric methods (e.g. Nanodrop) which can be unreliable to measure low DNA yield (Yu et al., 2017). Here, we present the results of a comparison of approaches for DNA recovery from buccal swabs in anurans across three case studies. Case Study 1 compares the efficiency of different types of buccal swabs, DNA extraction techniques and storage conditions for a widespread Eurasian anuran, the common toad (*Bufo bufo*). Case Study 2 compares the efficiency of different types of buccal swabs and extraction techniques in the widespread European common frog (*Rana temporaria*). Case Study 3 uses a multi‐species approach from Dominica and Madagascar, to compare different DNA preservation methods and swabbing regimes for samples collected in conditions less conducive to DNA preservation. Case Study 3 compares buccal swabs stored in different storage buffers, at different temperatures, and for different times between DNA collection and extraction. The results from these case studies aim to improve outputs obtained from a minimally invasive sampling technique and increase its application for genomic studies.

## 2 MATERIALS AND METHODS

Our study combines the results of buccal swabbing for genomic analyses from three anuran case studies: (1) single species *Bufo bufo*, (2) single species *Rana temporaria* and (3) multi‐species from Dominica and Madagascar. This work was undertaken as part of ongoing research studies that are not presented in this manuscript and were therefore originally collected for a purpose other than that described in this study.

### 2.1 Data collection

#### 2.1.1 Case Study 1 (*Bufo bufo*)

**FIGURE 1:**
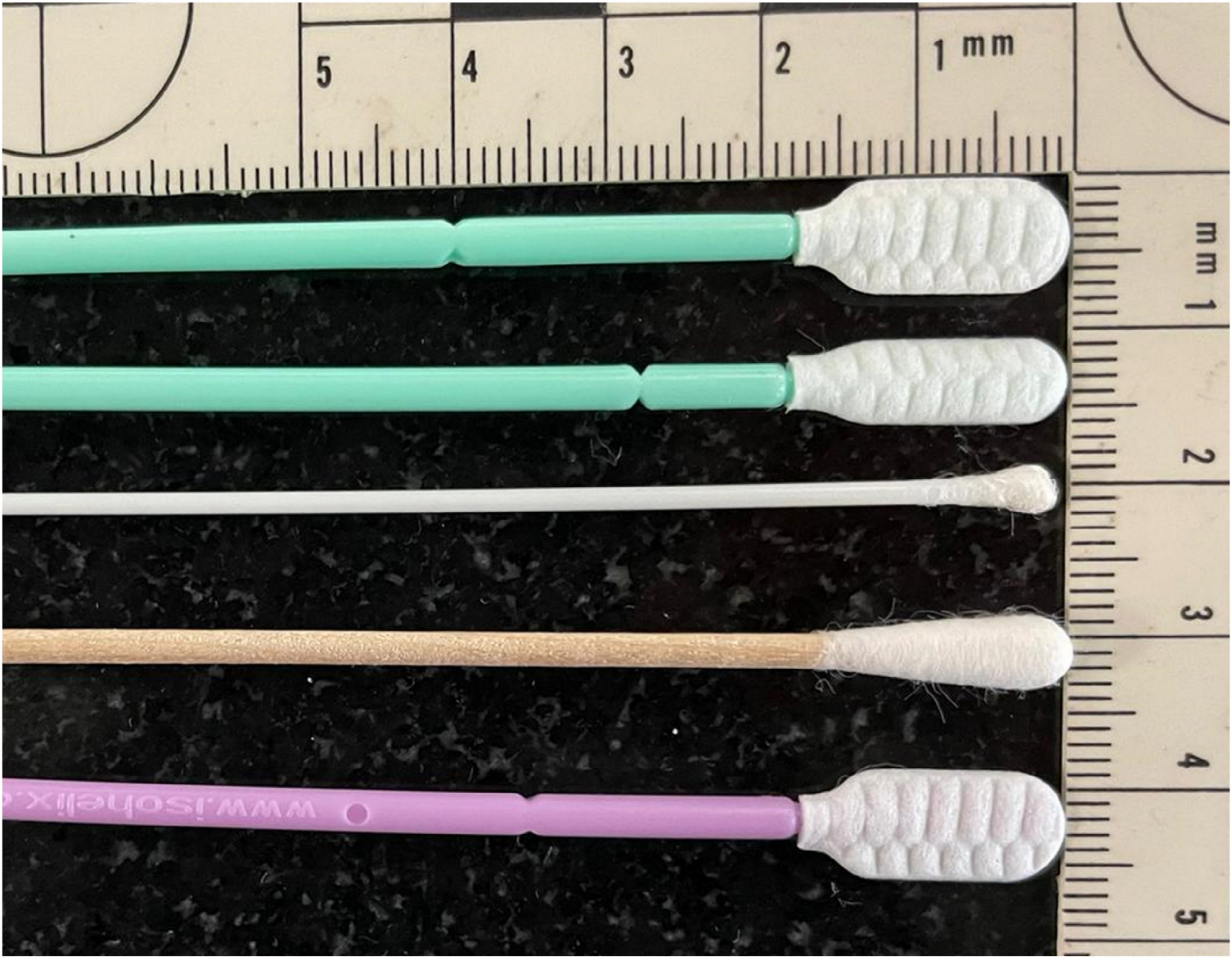
Types of swabs used in all case studies. From top to bottom: Isohelix™ SK‐3S (Case Study 1), Isohelix™ MS‐02 (Case Studies 1 and 2), rayon‐tipped MW113 fine‐tip (Case Studies 1 and 3), Wooden swab with large cotton tip (Case Studies 1 and 2), Isohelix™ Rapidry (Case Study 2).

Four different sterile swab types were trialled for DNA recovery using buccal swabbing in *B. bufo*: (i) wooden shafted swabs with large cotton tips (Technical Service Consultants Ltd.), (ii) Isohelix™ SK‐3S with plastic shafts and large flattened heads (Cell Projects Ltd.), (iii) Isohelix™ MS‐02 type swabs with plastic shafts and small flattened heads (Cell Projects Ltd.) and (iv) rayon‐tipped MW113 cotton swabs with plastic shafts (Medical Wire and Equipment) (Figure 1). Samples were stored in sterile 2 mL screw cap microcentrifuge tubes filled with 98% molecular‐grade ethanol or in a non‐alcoholic preservative buffer (10 mM Tris HCl, 5 mM EDTA, 0.5% SDS, pH = 7.8). Samples were stored at −80°C as soon as possible after collection until DNA extraction.

Swabbing was performed by handlers with previous experience in buccal swabbing amphibians. Mouth opening was achieved by sliding a small stick into the side of the mouth which caused the animal to open its mouth, after which the swab was inserted and rotated for 5 s in the buccal cavity, over and under the tongue, over the mucosal surface of the mouth cavity, making sure to avoid the underside of the eyes. See Data S1 for breakdown of swabbing protocol.

#### 2.1.2 Case Study 2 (*Rana temporaria*)

Three types of buccal swabs were tested for *R. temporaria*: (i) wooden swabs with a large cotton tip, (ii) Isohelix™ MS‐02 swabs stored in 98% molecular‐grade ethanol and (iii) Isohelix™ Rapidry swabs (Cell Projects Ltd.) that do not require storage in a preservative buffer after sample collection due to coming with a Rapidry pouch (Figure 1). Samples were stored at −80°C as soon as possible after collection until DNA extraction. The swabbing protocol was identical to Case Study 1.

#### 2.1.3 Case Study 3 (Madagascar and Dominica)

All samples for Case Study 3 were collected using sterile rayon‐tipped MW113 fine‐tip swabs. For Dominican taxa, buccal swabs were collected from mountain chicken frogs *Leptodactylus fallax* (*n* = 27) and Martinique robber frogs *Eleutherodactylus martinicensis* (*n* = 11). Buccal swabs were collected from several frog species in Madagascar (permit N^o^332/19/MEDD/SG/DGEF/DGRNE) including *Mantidactylus betsileanus* (*n* = 38), *Mantidactylus mocquardi* (*n* = 1), *Mantidactylus femoralis* (*n* = 1), *Anodonthyla vallani* (*n* = 1), *Guibemantis liber* (*n* = 1), *Platypelis pollicaris* (*n* = 1) and *Spinomantis peraccae* (*n* = 1). Mouth opening was achieved by gently sliding and rotating a very small spatula into the side of the mouth, and then the swab was inserted and rotated for 5 s over and under the tongue and over the mucosal surface of the mouth cavity, avoiding the underside of the eyes. Upon collection, swabs were stored in sterile 1.5 mL microcentrifuge tubes filled with 500 μL 95% ethanol, Longmire lysis buffer, or a sterile silicon dioxide (silica) capsule (Hypromellose capsule filled with moisture indicator 0.2–1 mm silica gel). For 1 L of Longmire lysis buffer, we used 100 mL 1 M Tris, 100 mL 1 M EDTA, 50 mL 10% SDS, 2 mL 5 M NaCl, 20 mL of 10% NaN3 and 728 mL H2O. Buccal swabs were stored at different temperatures (ambient: 20–25°C; fridge: 4°C; frozen: −20°C) and lengths of time (0.5–14 months), as described in Table 1.

**TABLE 1:** Summary table of buccal swab samples included in Case Study 3 (numbers of swabs for each condition).

	Temp	Months	Buccal swabs
Ethanol	Fridge	0.5	2
		1	1
		2	4
	Freeze	14	15
Longmire	Ambient	2	8
		3	21
	Freeze	5	1
		7	6
		8	4
		11	4
Silica	Fridge	0.5	1
		1	6
		2	9

### 2.2 DNA extraction and quantification

#### 2.2.1 Case Studies 1 and 2

DNA was extracted from swabs using two methods: (1) the animal tissue protocol of the Qiagen™ DNeasy Blood and Tissue Kit and (2) McHale's salting‐out DNA extraction protocol (OpenWetWare Contributors, 2009). Small amendments were made for both methods: we modified step 1 of the DNeasy Blood & Tissue Kit protocol by adding 300 μL of ATL buffer for bigger swabs (Isohelix Rapidry and SK‐3S), instead of the prescribed 180 μL, in order to ensure that the lysis buffer covered the entirety of the swab tip. The final elution volume was 100 μL of AE buffer. We modified the McHale's salting‐out extraction method as follows: (1) the incubation period of −20°C was expanded from 15 to 60 min; and (2) the washing of the pellet used ice chilled 70% and 100% ethanol (instead of non‐chilled). Pellets were then resuspended in a final elution of 100 μL of molecular‐grade distilled water. Following DNA extraction, the DNA concentration for each sample was measured for 237 *B. bufo* and 38 *R. temporaria* samples using a Qubit™ dsDNA Quantification High Sensitivity Assay Kit. DNA purity was measured with a Nanodrop 2000.

#### 2.2.2 Case Study 3

For Case Study 3, all 82 MW113 buccal swabs were processed using the Qiagen™ DNeasy Blood and Tissue Kit protocol for animal tissue. Swabs stored dry were transferred into microcentrifuge tubes filled with 180 μL of ATL buffer and 20 μL of proteinase K before incubation overnight at 56°C. Ethanol‐stored swabs were removed from the ethanol and allowed to dry at room temperature for 10 min before being transferred into microcentrifuge tubes filled with 180 μL of ATL buffer and 20 μL of proteinase K and incubated overnight at 56°C (as per manufacturer guidelines). Proteinase K was added directly to the Longmire lysis buffer and swab before incubation overnight at 56°C, and all of the solution was used in the subsequent steps. All DNA was eluted in 100 μL of AE buffer. DNA concentration was measured using a Qubit™ dsDNA Quantification High Sensitivity Assay Kit.

### 2.3 Mitochondrial DNA amplification, sequencing and processing

For all case studies, DNA extracts (or a subset in Case Study 1) were used to further test the efficiency of DNA preservation and extraction methods by performing PCR amplifications. A partial region of the mitochondrial rRNA (*16 s*) was targeted using polymerase chain reaction (PCR). The *16 s* primers 16SA‐L (5′‐CGC CTG TTT ATC AAA AAC AT‐3′) and 16SB‐H (5′‐CCG GTC TGA ACT CAG ATC ACGT‐3′) were used (Palumbi, 1996). Each PCR reaction consisted of 1 μL of extracted genomic DNA, 1 μL of each primer, 2 μL MyTaq™ Red Mix and 6 μL *dd*H_2_O for a total volume reaction of 12 μL. Amplification conditions were as follows: initial denaturation for 5 min at 94°C; 35 cycles of 30 s at 94°C, 30 s at 56°C and 1 min at 72°C; and a final extension of 5 min at 72°C. Success of amplified PCR reactions was assessed on a 1% TBE agarose gel.

Successfully amplified PCR products were prepared for sequencing. The PCR products were purified by adding 5 μL of PCR product with 2 μL of ExoSAP‐IT, incubated at 37°C for 15 min, followed by deactivation of the ExoSAP‐IT by heating to 80°C for 15 min. 0.5 μL BigDye V3.1 terminator, 2 μL 5x Terminator sequencing buffer and 0.5 μL of forward primer were added to the cleaned PCR products. Cycling conditions were as follows: 96°C for 2 min, then 25 cycles of 96°C for 10 sec, 52°C for 15 sec and 60°C for 3 min. Finally, 45 μL SAM solution and 5 μL of XTerminator Sequencing Buffer were added to the cleaned PCR products, wrapped in aluminium foil to protect the reagents from light and vortexed for 10 min. Tubes were then centrifuged for 2 min before 10 μL of each prepared sample was loaded onto a 96‐well plate. Samples were sequenced on the University of Wolverhampton in‐house ABI3500 Genetic Analyser.

Sequences were checked, cleaned and aligned using Geneious Prime v. 2023.1.2. Forward sequences were checked for any ambiguities and trimmed. Alignments were manually checked and cross‐referenced with raw sequences to account for any ambiguities. Species identity was confirmed by blasting to reference sequences in GenBank through Geneious Prime.

Additionally, for Case Study 1, samples were used for the genotyping of four microsatellite markers of *B. bufo* (Bb15, Bb39, Bb54, Bb62; Brede et al., 2001). PCRs were made with 6 μL of MyTaq™ Red Mix, 4 μL of molecular‐grade *dd*H_2_O, 0.5 μL of 10 ng/μL of each fluorescently labelled forward and reverse primer, and 1 μL of DNA extract for a total volume reaction of 12 μL. PCR conditions were 94°C for 5 min, followed by 40 cycles of 94°C for 30 s, a primer‐specific annealing temperature (see Brede et al., 2001) for 30 s and 72°C for 30 s, followed by a final step of 72°C for 5 min. PCR products were analysed with an ABI3500 Genetic Analyser and sized using Geneious Prime v. 2023.1.2.

### 2.4 Statistical analyses

We broadly compared DNA concentrations (from all types of swabs, storage conditions and extraction techniques) obtained from all case studies with a Kruskal–Wallis one‐way analysis of variance with Bonferroni correction. We used Wilcoxon signed‐rank tests to test for differences between DNA concentration between species in Case Studies 1 (*B. bufo*) and 2 (*R. temporaria*) because of the overall similarity in the study design of the two case studies. We also tested for differences in DNA concentration obtained from MW113 swabs between Case Studies 1 and 3 with a Wilcoxon signed‐rank test.

#### 2.4.1 Case Studies 1 and 2

DNA concentration was compared for different extraction methods and sample types using one‐way analysis of variance (ANOVA), with DNA concentration and purity as response variables and DNA extraction method, type of swab, and preservation procedure as explanatory variables. When assumptions for parametric tests could not be met (even following log or rank transformation of data), we used nonparametric equivalents (Wilcoxon signed‐rank tests for two group comparisons and Kruskal–Wallis one‐way analysis of variance with Bonferroni correction for more than two groups). Sample sizes of the different groups were too dissimilar to properly compare additive or interactive effects between explanatory variables. Since only a few swabs stored in non‐alcoholic buffer were extracted using the Qiagen™ method in Case Study 1, we compared the two extraction methods only for samples preserved in ethanol. For Case Study 2, wooden swabs consisted of only three samples extracted with the Qiagen™ method and these were therefore not included in extraction method analyses. Due to these limitations, we did not use model selection based on GLMs for the best predictor of DNA concentration between our variables.

All analyses were completed in *R* version 4.3.1 (R Core Team, 2023), with figures generated using the *ggplot2* package (Wickham et al., 2016). Results were considered significant when *p* < .05.

#### 2.4.2 Case Study 3

A general linear model (Gaussian family) was used to determine the impact of various storage conditions on DNA concentration. DNA concentration was the response variable, and factors varying in the collection or storage method were the explanatory variables. Explanatory variables were storage (dry, ethanol, Longmire, silica), temperature (ambient, fridge or frozen at −20°C), number of months stored and surveyor. An initial model, including all fixed terms, was built and minimised in a stepwise approach using the ‘drop one’ function in *R* version 4.2.3 (R Core Team, 2023). AIC values were compared between the models to select the final model. The initial model was *glm(log(conc)) ~ storage + temp + month + size + surveyor*. The final model run for analysis was *glm(log(conc)) ~ storage + temp + month*. The response variable concentration was transformed on a logarithmic scale using the log function to satisfy the assumption of normality in the model.

## 3 RESULTS

DNA concentration obtained from all swab types and extraction methods did not differ significantly between all samples from Case Studies 1 (*B. bufo*) and 2 (*R. temporaria*) (*W* = 4497, *p* = .98) but differed when including all case studies (*H* = 27.97, d.f = 2, *p* < .001). When only comparing concentrations obtained from the same type of swab between Case Studies 1 and 3 (MW113), we found no significant differences (*W* = 814.5, *p* = .5). Overall PCR amplification success (proportion of PCR reactions that led to a readable genotype) after one iteration for *16 s* was 95% for Case Study 1, 79% for Case Study 2 and 100% in Case Study 3 (Table 2). Small differences in the type of polymerase used and person handling the samples can explain differences in amplification success between case studies. Amplification success of microsatellites (Case Study 1) ranged from 86% to 96% across the four markers.

**TABLE 2:** Proportion of PCR amplification success for *16 s* after one iteration for the different types of swabs, storage techniques and extraction methods used in Case Studies 1 and 2.

	*16 s* amplification success (%)
*Case Study 1*	
Swab type	
SK‐3S (*n* = 3)	67
MS‐02 (*n* = 70)	96
MW113 (*n* = 4)	100
Wooden swab (*n* = 31)	94
Storage	
Ethanol (*n* = 87)	98
Non‐alcoholic (*n* = 21)	81
DNA extraction method	
Salting (*n* = 58)	93
Qiagen (*n* = 50)	96
*Case Study 2*	
Swab type	
MS‐02 (*n* = 15)	67
Rapidry (*n* = 20)	95
Wooden swab (*n* = 3)	100
Storage	
Ethanol (*n* = 18)	72
Dry (*n* = 20)	95
DNA extraction method	
Salting (*n* = 11)	82
Qiagen (*n* = 27)	78

### 3.1 Case Study 1

**FIGURE 2:**
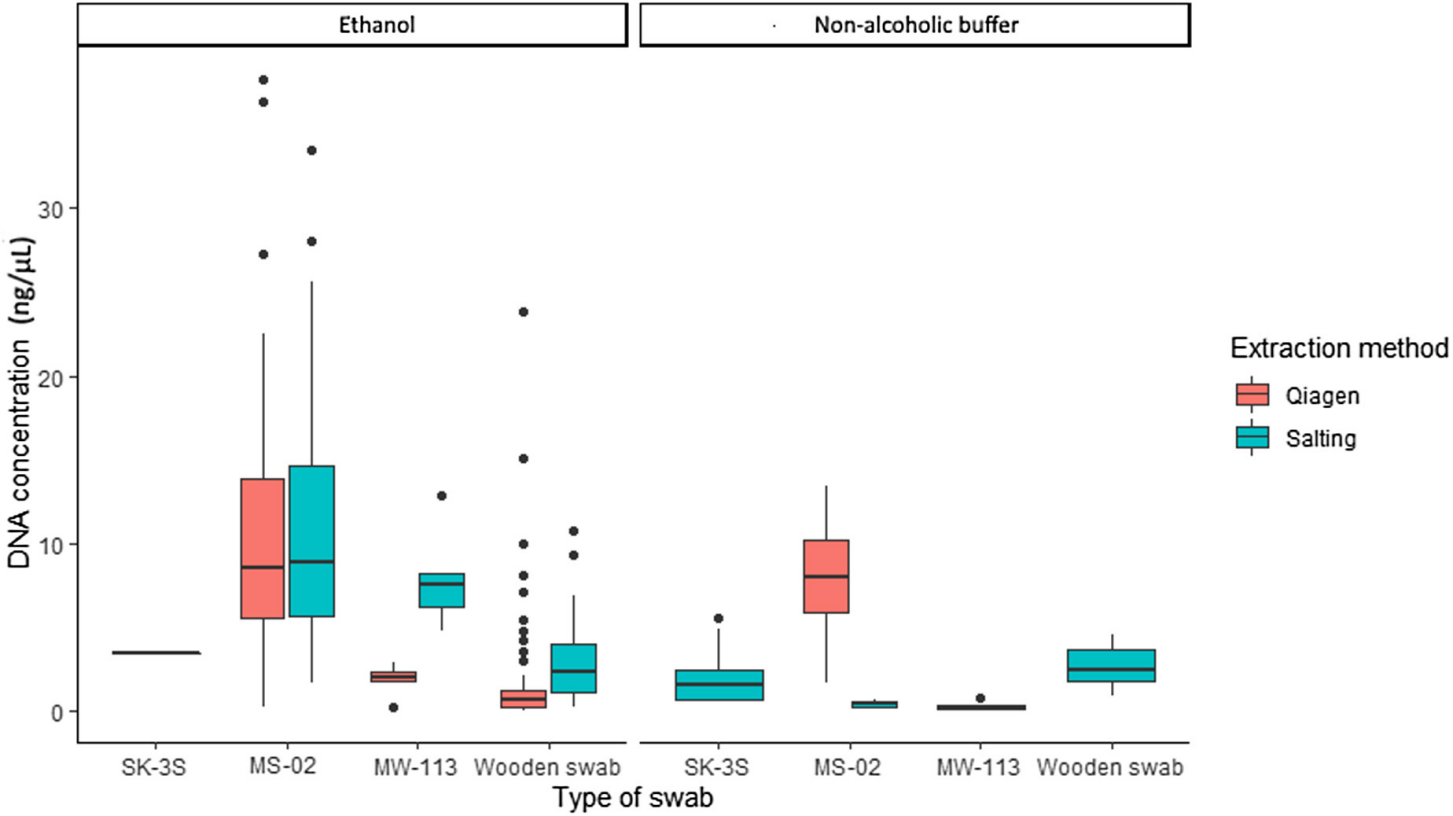
DNA concentration (ng/μL) obtained from four different types of swabs, preservation methods and extraction methods, for Case Study 1 (*Bufo bufo*) samples.

For *B. bufo*, mean DNA concentration extracted from each swab type varied from 2.19 ± 0.49 to 9.97 ± 0.74 ng/μL (Table 3), with the type of swab significantly influencing DNA concentration of extracts [*H*(3) = 90.46, *p* < .001]. MS‐02 swabs give greater yields compared to other types of swabs (Figure 2). Higher DNA yields were recovered from ethanol‐preserved swabs compared to non‐alcoholic buffer (*W* = 1997, *p* < .001; Table 2). The extraction method did not influence DNA recovery from MS‐02 swabs (*W* = 983, *p* = .83; Table 2); however, yields were significantly higher using the salting‐out extraction method for samples extracted from MW113 [*T*(9.32) = 1.89, *p* = .01] and wooden swabs (*W* = 481, *p* < .001; Figure 2).

**TABLE 3:** DNA concentration (ng/μL) of extracts obtained from *Bufo bufo* for different types of swabs, extraction methods and storage buffers.

Variable	Swab type SK‐3S	Swab MS‐02	Swab wooden	Swab MW113	Extraction method Qiagen™	Extraction method salting	Buffer ethanol	Buffer non‐alcoholic
Mean ± SE	2.19 ± 0.49	9.97 ± 0.74	2.34 ± 0.34	3.05 ± 0.84	5.44 ± 0.64	6.43 ± 0.59	6.29 ± 0.50	2.96 ± 0.57
*N*	12	105	102	18	120	117	200	37

**FIGURE 3:**
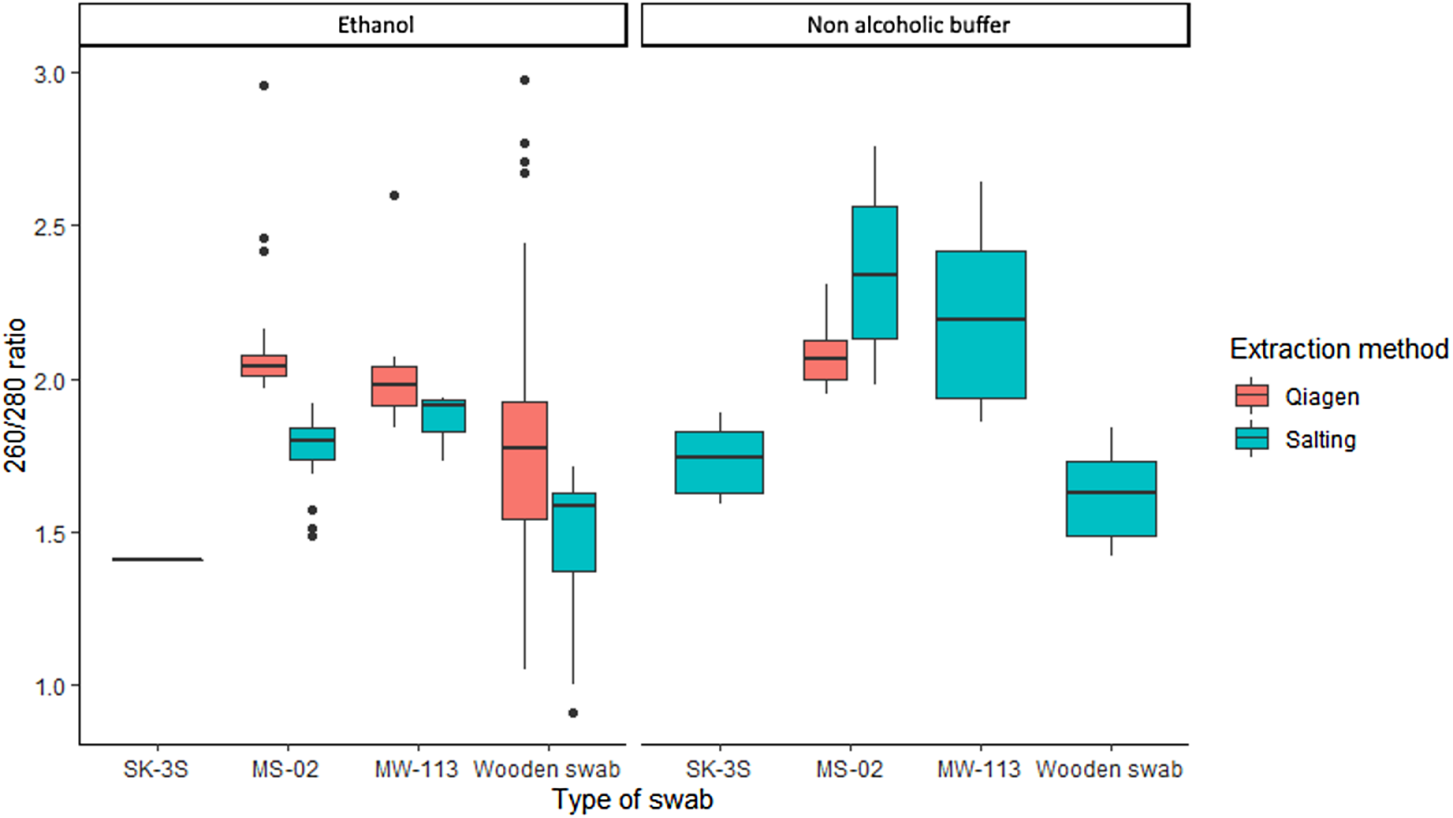
260/280 absorbance ratio obtained from four different types of swabs, preservation methods and extraction methods, for Case Study 1 (*Bufo bufo*) samples.

The type of swab influenced DNA purity as measured by 260/280 nm absorbance. Extracts obtained from wooden swabs had significantly lower purity than those from other swabs [*H*(3) = 93.73, *p* < .001; Figure 3]. Despite noticeable variation in absorbance, most extracts had a 260/280 value between 1.8 and 2 which is considered pure (Van Wieren‐De Wijer et al., 2009). Samples extracted using the Qiagen™ method had higher 260/280 ratios compared to samples extracted with the salting‐out method (*W* = 7434, *p* < .001; Figure 3). Extract purity was significantly different for each buffer type (*W* = 806, *p* = .01) with a mean 260/280 ratio of 1.66 for samples preserved in ethanol and a mean 260/280 ratio of 1.88 for the non‐alcohol buffer‐preserved samples, suggesting better purity for samples extracted from the latter. However, when the wooden swabs were removed from the analyses, mean 260/280 was 1.78 for ethanol‐preserved and 1.98 for non‐alcoholic buffer‐preserved samples, suggesting that extracts from both methods fall into the edges of pure DNA range for 260/280 nm absorbance.

### 3.2 Case Study 2

**FIGURE 4:**
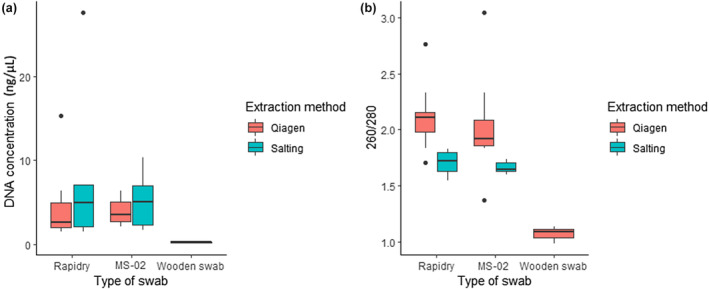
DNA concentration (ng/μL) obtained from three types of buccal swabs and two extraction methods (a), and 260/280 absorbance ratio obtained from three types of swabs and two extraction methods (b), for *Rana temporaria* samples.

For *R. temporaria*, no differences in DNA recovery were observed between the MS‐02 and Rapidry swabs (*W* = 121.5, *p* = .35; Figure 4a) with mean DNA concentration of 4.49 ± 0.64 and 5.14 ± 1.38 ng/μL, respectively (Table 4). DNA concentration was not significantly different between extraction methods (*W* = 104.5, *p* = .16; Table 4).

**TABLE 4:** DNA concentration (ng/μL) of extracts obtained from *Rana temporaria* for different types of swabs and extraction methods.

Variable	Swab type Rapidry	Swab MS‐02	Swab wooden	Extraction method Qiagen™	Extraction method salting
Mean ± SE	5.14 ± 1.38	4.49 ± 0.64	0.22 ± 0.05	3.56 ± 0.57	6.78 ± 2.26
*N*	20	15	3	27	11

For *R. temporaria* samples, wooden swabs had lower purity than other swabs [*F*(2) = 15.71, *p* < .001], while MS‐02 and Rapidry swabs did not differ significantly [*T*(34) = 1.53, *p* = .14]. Extraction method influenced the 260/280 ratio [*T*(32) = 6.17, *p* < .001] with the Qiagen™ extraction method having higher 260/280 absorbance ratios (Figure 4b).

### 3.3 Case Study 3

**FIGURE 5:**
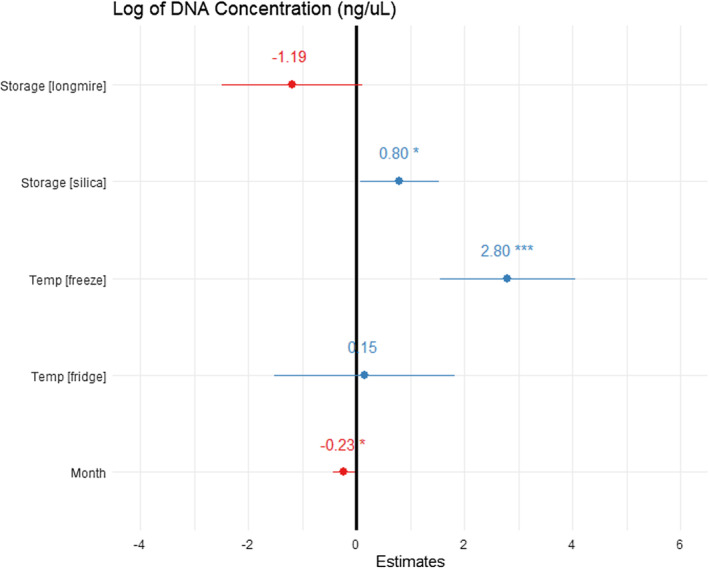
Effect sizes (variation in parameters estimates with 95% confidence interval) of storage conditions on DNA concentration yielded from buccal swabs for several Madagascan frog species as determined by a GLM. The black vertical line indicates no effect. Storage buffer Longmire lysis and silica are compared to ethanol. Storage temperature for all the above are grouped into fridge or freeze and compared to ambient temperature. Blue lines show positive effects, red lines show negative effects. * Indicates significative effect with *p* < .05 and *** indicates a significative effect with *p* < .001.

The general linear model recovered DNA concentration as significantly associated with storage type, length of time stored (months), and temperature (adjusted *R* ^2^ = 0.5576, *F*_5,76_ = 21.41, *p* < .001; Figure 5). Storage in Longmire buffer yielded lower concentrations of DNA than in ethanol or silica, but this was not significant. When stored with silica pellets, significantly higher concentrations of DNA were recovered when compared to ethanol‐preserved swabs (0.795 ± 0.36 ng/μL, t = 2.185, *p* = .03). Freezing yielded significantly more DNA than from swabs stored refrigerated or at ambient temperature (2.80 ± 0.63 ng/μL, *t* = 4.435, *p* < .05). Refrigerated swabs yielded more DNA than those stored at ambient temperature but not significantly. Increase in storage time significantly decreased the concentration of recovered DNA (−0.232 ± 0.1 ng/μL, *t* = −2.305, *p* = .02).

## 4 DISCUSSION

Our results demonstrate that buccal swabs of anurans can yield enough DNA of good purity that can be used for downstream genetic applications. Our results highlight that the type of swab used, the preservation strategy and the extraction method can significantly impact overall DNA recovery (yield and purity). Some sampling strategies may be more appropriate than others under differing field collection conditions, and thus careful consideration is required to optimise DNA recovery when using buccal swabs. While downstream use was not a focus of this study, our methods were robust for generating data for traditional (Sanger and microsatellite) and high‐throughput (low‐coverage whole‐genome sequencing and ddRAD‐seq) molecular approaches (Martin et al., *unpub. data*; Mullin et al., *unpub. data*).

When comparing DNA extracts from two similar‐sized amphibian species (*B. bufo* and *R. temporaria*), DNA concentrations did not differ significantly, supporting the robustness of the different methods reported herein. However, the type of swab significantly impacted DNA yield, with Isohelix™ MS‐02 and Rapidry being the most effective swabs. Lower concentration and lower purity of DNA were obtained from wooden swabs making these the least efficient type of swab. Overall, drying samples with silica is a better preservative technique than using ethanol (Case Study 3), while ethanol is a better preservative than non‐alcoholic buffer (Case Study 1). The salting‐out extraction method is better for recovering greater yields of DNA, but the Qiagen™ extraction method gives higher purity. Storage conditions (temperature and time to extraction) are also important to guarantee high DNA recovery following extraction, with freezing being the best approach, because DNA recovery from swabs decreases with increased time between sample collection and extraction.

Our results indicate that the type of swab is important in determining the yield and purity of recovered DNA. Shape and size of the head of the swab will impact the ability to use them for small vertebrates, and depending on the body and mouth size of the target species, the optimal swab will differ. For medium‐sized anurans, the flattened and ridged head of MS‐02 and Rapidry swabs were more effective at collecting DNA than the rounded shape of the cotton tip of the wooden and MW113 swabs. However, MS‐02 and Rapidry swabs may be too large for small species.

Storage buffer also influenced DNA concentration, with ethanol being better than our non‐alcoholic preservative buffer. Ethanol is not always readily available and may not be a suitable option for transportation by plane or post. Storing samples dry represents a useful short‐term preservation method, as evidenced by the dry Rapidry swabs recovering similar yields and purity to samples from MS‐02 swabs stored in ethanol. Silica also proved to be a better alternative compared to ethanol to guarantee higher DNA recovery. Silica helps to actively dry the sample, preventing enzymes from breaking down DNA which can explain our results (Michaud & Foran, 2011). Our results are similar to those of Colussi et al. (2017) who found that in rainbow trout (*Oncorhynchus mykiss*), dried buccal swabs yielded higher DNA concentration than ethanol‐ or PBS‐preserved samples. Our results confirm the earlier findings of Broquet et al. (2007) and Pidancier et al. (2003) that cooler temperatures improve DNA preservation because increased temperatures can negatively affect the stability of DNA (Kasai et al., 2020). While the ultimate recommendation for freezing DNA remains, we have demonstrated that any reduction in temperature is beneficial for DNA preservation. Every effort should be made to keep samples in the shade and a cool box if possible when samples are collected in the field and refrigeration/freezing appliances are not available. It is, however, worth noting that we have been able to successfully perform mtDNA barcoding (Mullin et al., 2021, 2022; Rakotoarison et al., 2023) and GBS/ddRAD‐seq (Mullin, *unpub. data*) from samples stored at ambient, tropical temperatures.

Additionally, in Case Study 1, DNA extraction method had an impact on DNA concentration depending on the type of swab. Extraction method affected DNA yield for samples collected with MW113 and wooden swabs but not for MS‐02. The differences observed may be due to the amount of salt remaining in solution after the extraction procedure. Differences in salt concentration are also likely to impact Qubit™ measurements, because low levels of salt will change DNA structure and impact the accuracy of fluorimetric‐based quantification (Nakayama et al., 2016).

The type of swab, preservative buffer, and extraction method used each influenced DNA purity (260/280 absorbance). A 260/280 value between 1.8 and 2 is considered clean DNA, and only the DNA extracted from wooden swabs consistently fell below this range, possibly indicating protein contamination (Van Wieren‐De Wijer et al., 2009). Differences in DNA purity between extraction methods may be linked to salt content in final extracts, as well as possible variation in pH. The salting‐out extraction method seems more prone to protein contamination, whereas higher absorbance obtained with the Qiagen™ protocol is more likely to result in co‐extraction of RNA and DNA, which could explain absorbance values >2.0 in some swabs extracted with the Qiagen™ method (Figures 3 and 4b).

A major and often overlooked aspect of DNA collection from buccal swabs is the standardisation of the swabbing technique and personal experience of the collector. Several different collectors were involved in our sample collection and, although all were experienced, small individual differences in swabbing procedures (exact time and swabbing technique) still remain. Variation in buccal cavity size of individual animals could also affect the amount of DNA collected. Food or water consumption shortly before sampling can possibly alter DNA recovery as some food items or soil ingested during prey capture may contain PCR inhibitors (Bessetti, 2007). For Case Studies 1 and 2, most samples were taken during the breeding season, a period in which individuals feed less than other active periods. Although we stored samples at −80°C (Case Studies 1 and 2) or −20°C (Case Study 3) as soon as possible after collection, we could not standardise the exact amount of time between collection and storage in Case Studies 1 and 2, and therefore cannot evaluate possible differences in DNA degradation between samples prior to extraction. Although Case Study 3 highlighted that storage conditions (e.g. temperature and time to extraction) impact DNA recovery, we consider our results for Case Studies 1 and 2 to be robust due to the number of samples obtained for each of the preservation methods used.

Our results provide compelling support that buccal swabs are reliable alternatives to traditional, destructive tissue collection methods for obtaining high yields and good‐quality DNA for genomic applications, as supported by other studies (Ambu & Dufresnes, 2023; Broquet et al., 2007). We demonstrate that the choice of swab, preservation approach and extraction method should be carefully considered when performing genetic studies in order to maximise DNA yield and purity. In field‐based studies, and with funding limitations, optimal conditions are almost never achievable; therefore, trade‐offs between cost, time and efficacy need to be made (see 4.1 Recommendations).

Standardised methods for buccal swabbing are important to ensure that enough clean genetic material is collected while reducing animal handling time to limit stress. Providing detailed swabbing procedure can hopefully help reduce the variability of DNA yields recovered from different swabbers and reduce the issue of ‘gentle swabbing’, by which samplers fail to collect enough genetic material due to an overly cautious swabbing method, or on the contrary causing damage or stress to the animals in question by being too rough in handling and vigorous in swabbing. We present a detailed information guide about buccal swabbing procedure in anurans Data S1. These recommendations are similar to the swabbing method used by Ambu and Dufresnes (2023) but adapted to our protocol and sampling strategy.

To help practitioners in choosing the best approach for their study design, we calculated the estimated cost per sample of each type of swab, storage technique and extraction method (Table 5). By highlighting the importance of study design for DNA collection, which is often overlooked in genetic acquisition studies, our results should act as a guide and help improve practices for genetic collection of samples from vertebrates, towards less invasive, but efficient, DNA collection procedures.

**TABLE 5:** Estimated cost per sample (GBP £) of each type of swab, storage technique and DNA extraction methods used in all case studies.

Swab type					Storage method			Extraction procedure	
Wooden	MW113	MS‐02	SK‐3S	Rapidry	Ethanol	Non‐alcoholic/Longmire	Silica	Qiagen™ DNeasy Blood and Tissue kit	McHale's salting out
£0.2	£0.32	£1.24	£1.08	£1.66	£0.13	£0.02	£0.06	£3.79	£0.62

*Note*: For swabs, the cost includes the price of one swab and the estimated cost of a 1.5 mL Eppendorf for sample storage (est. £0.058), except for Rapidry swabs, as the drying pouch is provided. The estimated cost of the storage buffers was calculated on the basis of 1 mL buffer per sample. Prices were estimated based on prices obtained in December 2023.

### 4.1 Recommendations for collecting and extracting DNA from buccal swabs

**FIGURE 6:**
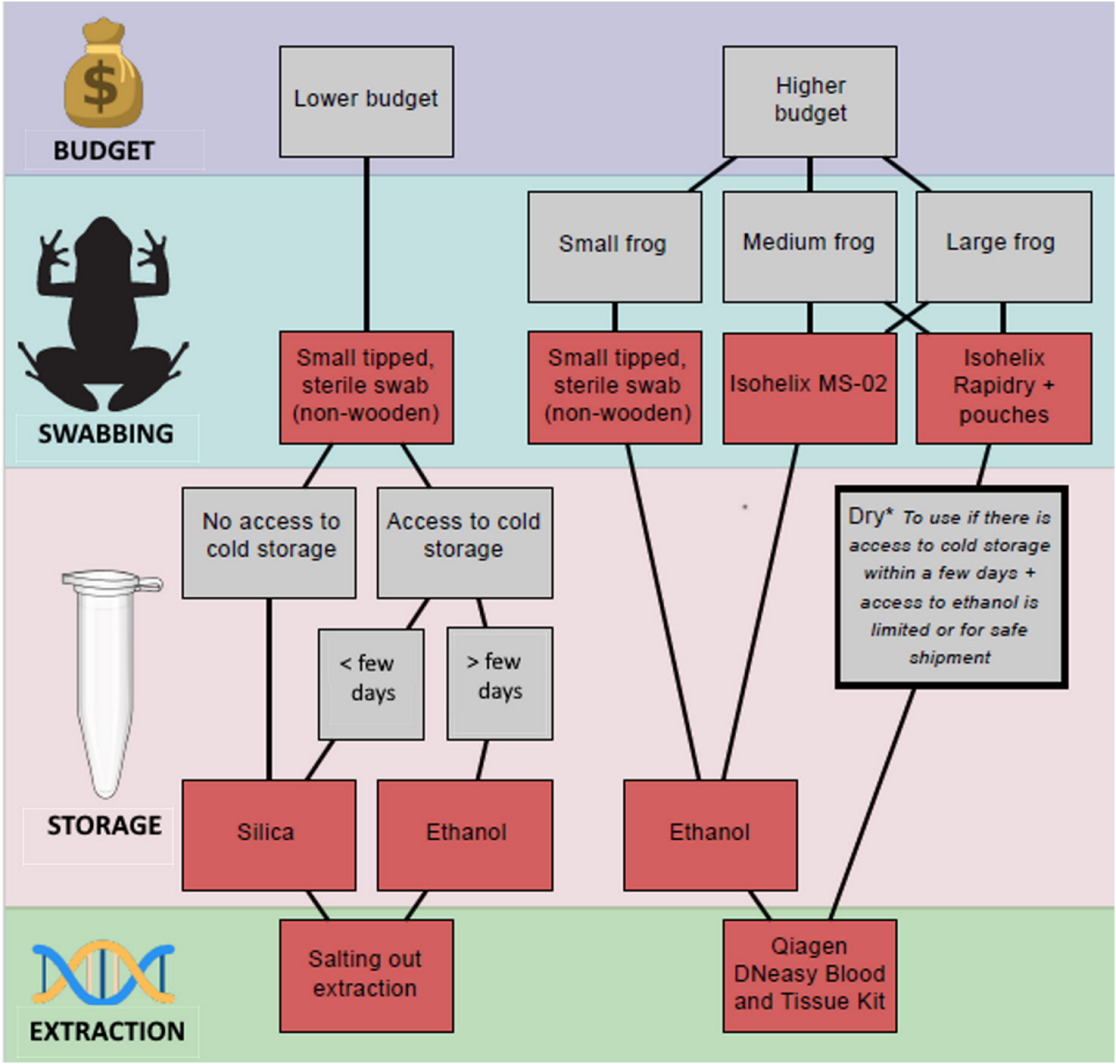
Summary of recommendations for selecting swab type, storage technique and DNA extraction method to improve DNA recovery from buccal swabs.

Based on our results, we recommend the use of Isohelix™ MS‐02 and Rapidry swabs for DNA collection of medium‐ to large‐sized anurans (see Figure 6 for a summary of our recommendations). The cost of these swabs is higher than many other commercially available swabs, but the DNA recovery means that the optimal DNA recovery is obtained. MW113 swabs are more adapted for small‐size species and can be an alternative to Isohelix™ swabs for researchers with a more limited budget or smaller species. Non‐alcoholic buffers are a cheaper but less reliable option than ethanol, but a variety of other homemade or commercially available preservative buffers were not tested here (e.g. RNAlater, DNA shield). It is important to note that non‐alcoholic buffers may be the best alternative for sample preservation due to limitations and regulations for transport and shipment of ethanol (IATA, 2023). Alternatively, dry storage such as for Isohelix™ Rapidry swabs and silica drying can be used as a short‐term solution if kept cool shortly after collection. Although DNA extraction technique did not influence final DNA yields, Qiagen™ DNeasy Blood and Tissue kits offer the advantage of a simpler and quicker extraction protocol compared to the modified McHale's salting‐out extraction method, however, the latter can be homemade and thus vastly less expensive (Table 5). Temperature conditions are also important for short‐term storage and transportation of samples, and emphasis should be given to keeping samples as cool as possible, ideally frozen, but avoiding multiple freeze–thaw cycles. Given our recommendations (Figure 6), where funding is not as limited, and assuming the sampling of medium/large vertebrates, we estimate that as of December 2023, per genetic sample it would cost ca. GBP£5.16 for sample collection and extraction. Our recommendations for lower budgets on the same date would equate to a cost of ca. GBP£1.00 for sample collection and extraction.

## AUTHOR CONTRIBUTIONS

**R. Martin:** Conceptualization (equal); data curation (lead); formal analysis (lead); funding acquisition (supporting); investigation (equal); methodology (equal); project administration (supporting); resources (equal); software (lead); supervision (equal); validation (equal); visualization (equal); writing – original draft (lead); writing – review and editing (lead). **K. E. Mullin:** Conceptualization (equal); data curation (supporting); formal analysis (supporting); funding acquisition (supporting); investigation (equal); methodology (equal); project administration (supporting); resources (equal); software (supporting); supervision (supporting); validation (equal); visualization (supporting); writing – original draft (supporting); writing – review and editing (equal). **N. F. D. White:** Conceptualization (equal); data curation (supporting); formal analysis (supporting); funding acquisition (supporting); investigation (equal); methodology (equal); project administration (supporting); resources (equal); software (supporting); supervision (supporting); validation (equal); visualization (supporting); writing – original draft (supporting); writing – review and editing (equal). **N. Grimason:** Conceptualization (supporting); data curation (supporting); formal analysis (supporting); funding acquisition (supporting); investigation (equal); methodology (supporting); project administration (supporting); resources (equal); software (supporting); supervision (supporting); validation (supporting); visualization (supporting); writing – original draft (supporting); writing – review and editing (equal). **R. Jehle:** Conceptualization (equal); data curation (supporting); formal analysis (supporting); funding acquisition (equal); investigation (supporting); methodology (supporting); project administration (supporting); resources (supporting); software (supporting); supervision (supporting); validation (equal); visualization (supporting); writing – original draft (supporting); writing – review and editing (equal). **J. W. Wilkinson:** Conceptualization (supporting); data curation (supporting); formal analysis (supporting); funding acquisition (equal); investigation (supporting); methodology (supporting); project administration (supporting); resources (supporting); software (supporting); supervision (equal); validation (equal); visualization (supporting); writing – original draft (supporting); writing – review and editing (equal). **P. Orozco‐terWengel:** Conceptualization (supporting); data curation (supporting); formal analysis (supporting); funding acquisition (equal); investigation (supporting); methodology (supporting); project administration (supporting); resources (supporting); software (supporting); supervision (supporting); validation (supporting); visualization (supporting); writing – original draft (supporting); writing – review and editing (equal). **A. A. Cunningham:** Conceptualization (supporting); data curation (supporting); formal analysis (supporting); funding acquisition (supporting); investigation (supporting); methodology (supporting); project administration (supporting); resources (supporting); software (supporting); supervision (supporting); validation (supporting); visualization (supporting); writing – original draft (supporting); writing – review and editing (supporting). **S. T. Maddock:** Conceptualization (equal); data curation (supporting); formal analysis (supporting); funding acquisition (lead); investigation (supporting); methodology (equal); project administration (lead); resources (supporting); software (supporting); supervision (lead); validation (lead); visualization (equal); writing – original draft (supporting); writing – review and editing (lead).

## CONFLICT OF INTEREST STATEMENT

Despite making recommendations on commercially available products, this was done without preliminary accord or interference from the companies producing these products. Therefore, the authors declare no conflict of interest.

### OPEN RESEARCH BADGES

This article has earned an Open Data badge for making publicly available the digitally‐shareable data necessary to reproduce the reported results. The data is available at https://doi.org/10.5281/zenodo.11203693.

## Supporting information


Data S1:

## Data Availability

All data generated or analysed during this study are included in this published article, its supplementary information files and publicly available repositories at: https://doi.org/10.5281/zenodo.11203693.
