# Supplementary material for: Optimising recovery of DNA from minimally invasive sampling methods: Efficacy of buccal swabs, preservation strategy and DNA extraction approaches for amphibian studies
Source: Ecol Evol. 2024 Sep 12;14(9):e70294. doi: 10.1002/ece3.70294 (PMC11392594; doi:10.1002/ece3.70294)
Supplement: Supplementary file 1 — Data S1: [file ECE3-14-e70294-s001.doc]

**Supplementary material S1**

**Amphibian buccal swabbing protocol**

**List of equipment**

- Gloves to avoid genetic contamination
- Fine pipet tip, small spatula or equivalent for mouth opening
- Lighter or alcohol wipe for equipment sterilization
- Buccal seab and appropriate strorage (Eppendorf with preservative buffer, silica capsule or storage pouch)
- Small bag to keep used gloves and used swabs wrapping and plastic shafts.

**Important points about sampling**

It is crucial to avoid contamination of genetic samples where possible. Therefore, when taking genetic samples always wear clean and fitted rubber gloves and use sterile equipment (spatula, scissors). If equipment drops on the floor or comes into contact with an unsterile object, sterilise it again (*see below for sterilisation instructions*); if a swab drops on the floor, use a new one.

*STERILISATION PROCEDURE:* Flame (with a cigarette lighter) metal equipment for 2 – 3 seconds before the first sample, between samples, and after the last sample. It is important to ensure that equipment is properly cooled before using any sterilised equipment. Alternatively, using alcohol and wipes can be used to sterilise equipment.

All steps shouldn’t take more than 1 – 2 mins to avoid overheating and prolonged stress on the animal. All handling should be completed by a competent and experienced handler.

**Protocol for sampling**

• Buccal swabbing can most easily be carried out by two sampling personnel, one handling the animal and maintaining the mouth open, and the other performing the swabbing. The protocol can however be completed by one experienced swabber. If the swabbing is done by one person, we recommend, prior to opening the mouth of the animal, to open the buccal swab packaging, making the shaft accessible, and leaving the tip inside to avoid contamination. That way, when the mouth is open, it is easy to get the swab ready to be inserted into the mouthf you are using Isohelix™ Rapidry swabs, it is recommended to open the storage pouch for the swab prior to opening the animal’s mouth.

• Safely restrain the animal firmly but without applying damaging pressure. For anurans, turn the animal to have its dorsum against the palm of your hand, whilst still maintaining a delicate but firm grip to prevent it from struggling. With a sterile pipet tip, small spatula or equivalent (avoid hard and pointy items), open the mouth by applying gentle pressure at approximately a 90 degree angle from below, pushing between the slightly protruding upper jaw and lower jaw at the anterior-most point of the mouth. When the tip is slightly inserted at the front of the mouth, slide/move the tip/spatula slightly to one side of the mouth until it reaches the side of the mouth and then insert it so that it sits across the mouth (similar to a horse snaffle) (Fig 1). It is critical to be delicate when performing these steps to avoid damaging the mouth. Some swabs (e.g., MW-02, Rapidry) are designed in such a way that there is no need to use an additional tool to open the mouth and instead the swab itself can be used for this.

• Meanwhile, the second swabber opens the sterile buccal swab wrapper (already done if the swabber is alone) and holds the shaft of the swab approximately half way along the shaft. Insert the swab into the mouth of the toad. The swab should not be inserted too deep into the buccal cavity (only the tip) (Fig 2).

• Remove the tip/spatula from the mouth when the swab has been inserted.

•Rotate the swab gently in the mouth, around the tongue and sides of the buccal cavity for approx. 10 sec, avoiding the eye socket (and teeth in the case of cecilians).

• After the swab has been removed from the mouth:

- If using a tube containing a preservative buffer: open the tube, push the swab tip to the bottom of the tube, snap the shaft if using a wooden swab or Isohelix™ swabs by pushing sideway against the inside of the tube (Fig 3); if using MW113 swabs use scissors to cut the shaft. Close and shake the tube to get the buffer in contact with the whole swab tip. If using a tube with silica (dry storage): shake the swab a few times to pre-dry the swab, insert the swab in the tube and close it. If the swab is too wet, it may not dry properly and start to rot.
- If using a Isohelix™ Rapidry swab: shake the swab gently a few times to pre-dry the swab, open the storing pouch if not done prior to the mouth opening, insert the swab in the pouch and seal with a sticker.

• When the tube/storage pouch is securely closed, release the animal where it was collected.

• Remove gloves after processing and put them in a trash bag or bin. Put new gloves on ready for processing the next individual.

• Use a permanent marker to write a unique sample identifier on either the collection tube or storage pouch.

If using storage buffer, it is better to always keep the tube upright to maintain the swab tip is submerged in the buffer during subsequent transport/storage of the genetic sample.

Freezer storage is recommended as soon as possible after sample collection, to avoid DNA degradation, especially for dried samples, as swab that are insufficiently dried may rot. However, once frozen, it is important to limit freeze-thaw cycles as much as possible.


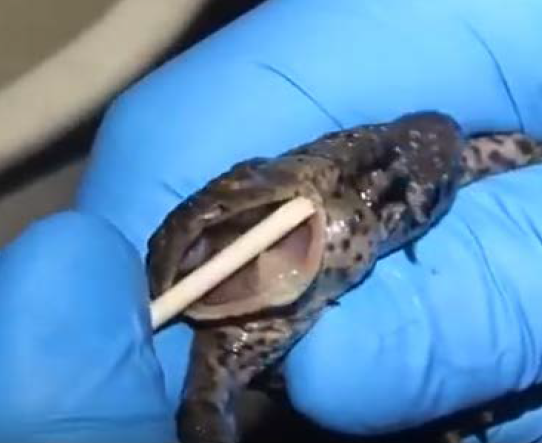


**Figure 1**: Mouth opening a *Bufo bufo* prior to inserting the swab in the buccal cavity


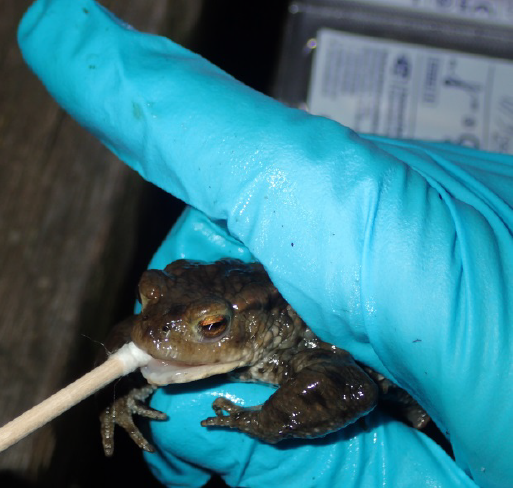


**Figure 2**: Swabbing a *Bufo bufo* while only inserting the tip of the swab in the mouth.


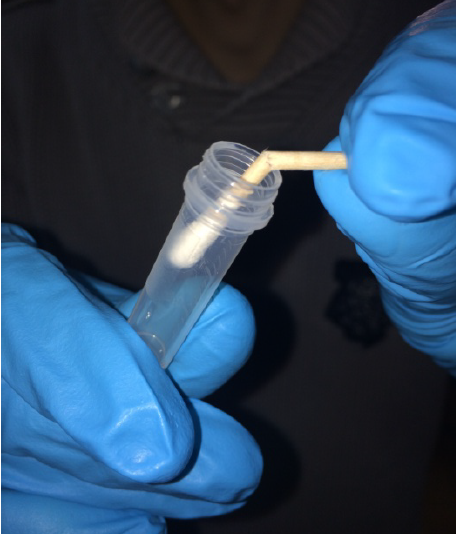


**Figure 3**: Storage of the swab tip by snapping the shaft against the side of the storage tube.
